# Fat fraction quantification with MRI estimates tumor proliferation of hepatocellular carcinoma

**DOI:** 10.3389/fonc.2024.1367907

**Published:** 2024-04-11

**Authors:** Mengqi Huang, Fan Zhang, Zhen Li, Yan Luo, Jiali Li, Zixiong Wang, Liya Ma, Gen Chen, Xuemei Hu

**Affiliations:** Tongji Hospital, Tongji Medical College, Huazhong University of Science and Technology, Wuhan, Hubei, China

**Keywords:** hepatocellular carcinoma, fat fraction, proliferation, MVI, MRI

## Abstract

**Purpose:**

To assess the utility of fat fraction quantification using quantitative multi-echo Dixon for evaluating tumor proliferation and microvascular invasion (MVI) in hepatocellular carcinoma (HCC).

**Methods:**

A total of 66 patients with resection and histopathologic confirmed HCC were enrolled. Preoperative MRI with proton density fat fraction and R_2_* mapping was analyzed. Intratumoral and peritumoral regions were delineated with manually placed regions of interest at the maximum level of intratumoral fat. Correlation analysis explored the relationship between fat fraction and Ki67. The fat fraction and R_2_* were compared between high Ki67(>30%) and low Ki67 nodules, and between MVI negative and positive groups. Receiver operating characteristic (ROC) analysis was used for further analysis if statistically different.

**Results:**

The median fat fraction of tumor (tFF) was higher than peritumor liver (5.24% vs 3.51%, P=0.012). The tFF was negatively correlated with Ki67 (r=-0.306, P=0.012), and tFF of high Ki67 nodules was lower than that of low Ki67 nodules (2.10% vs 4.90%, P=0.001). The tFF was a good estimator for low proliferation nodules (AUC 0.747, cut-off 3.39%, sensitivity 0.778, specificity 0.692). There was no significant difference in tFF and R2* between MVI positive and negative nodules (3.00% vs 2.90%, P=0.784; 55.80s-1 vs 49.15s-1, P=0.227).

**Conclusion:**

We infer that intratumor fat can be identified in HCC and fat fraction quantification using quantitative multi-echo Dixon can distinguish low proliferative HCCs.

## Highlights

- The intratumor fat on MRI is commonly detected and serves as an important ancillary feature for the diagnosis of HCC.
- The intratumor fat can be identified in HCC with chronic hepatitis and distinguish low proliferative HCCs which hold promise as a prognostic predictor.

## Introduction

Tumor proliferation and microvascular invasion (MVI) in hepatocellular carcinoma (HCC) are pivotal factors leading to early recurrence following radical therapy and portend a poor prognosis (1). The immunohistochemical marker Ki-67 serves as a common indicator of cell proliferation and has been established as an independent predictor of early recurrence and poor prognosis in surgically resected HCC (2). A preoperative evaluation of proliferation and MVI may furnish additional insights for treatment strategies, such as limitation of ablation and liver transplantation, neoadjuvant and adjuvant therapeutics. However, determining proliferation and MVI is currently confined to specimens, limiting their broader clinical application.

The intra-tumoral fat is commonly identified in HCC, manifesting in various degrees of differentiation (3). Fatty change is known to be a marker of transformation from dysplastic nodule to HCC. It is more prevalent in well-differentiated HCC, with focal fatty changes observed in larger tumors. In clinical practice, poorly differentiated HCC often exhibits focal fatty change. A prevalent hypothesis for intra-tumoral fatty change involves reduced blood flow due to portal tract destruction and inadequate neovascularization (4, 5), which exposes the HCC to a hypoxic environment. Concurrently, upregulation of hypoxia-related signaling pathways promote the proliferation and migration of cancer cells (6). Therefore, quantifying fatty changes may serve as an indicator of HCC proliferation. Additionally, the invasiveness of HCC is corelated with increased tumor vascularity, and the HCC with diffuse fat tends to grow slowly and with lower risk of MVI (7), leading to favorable prognosis (8). Nevertheless, the relationship between the risk of MVI and the fat quantification in HCC with focal fat and chronic hepatitis remains unclear.

MRI with multi-echo Dixon and chemical shift encoded technique possesses a unique advantage in evaluating intra-cellular fat, as it can distinguish signals of water, triglyceride (9), and iron (10), which provide a non-invasive and quantitative evaluation. The quantitative multi-echo Dixon enables iron correction and accurate detection of fat, as well as MRI spectroscopy (11, 12). The intra-tumoral fat on MRI is an important ancillary feature for diagnosing HCC (13) and a potential biomarker for favorable performances after treatment (14). Thus, we hypothesize that the quantitative multi-echo Dixon may enable preoperative, non-invasive, and comprehensive assessment of cell proliferation and MVI, which may guide clinical treatment selection. Hence, this study aims to estimate the diagnostic value of fat fraction quantification based on MRI quantitative multi-echo Dixon for tumor proliferation and MVI in HCC.

## Materials and methods

This retrospective study was approved by the institutional ethics committee and the requirement of informed written consent was waived.

### Patients

**Figure 1:**
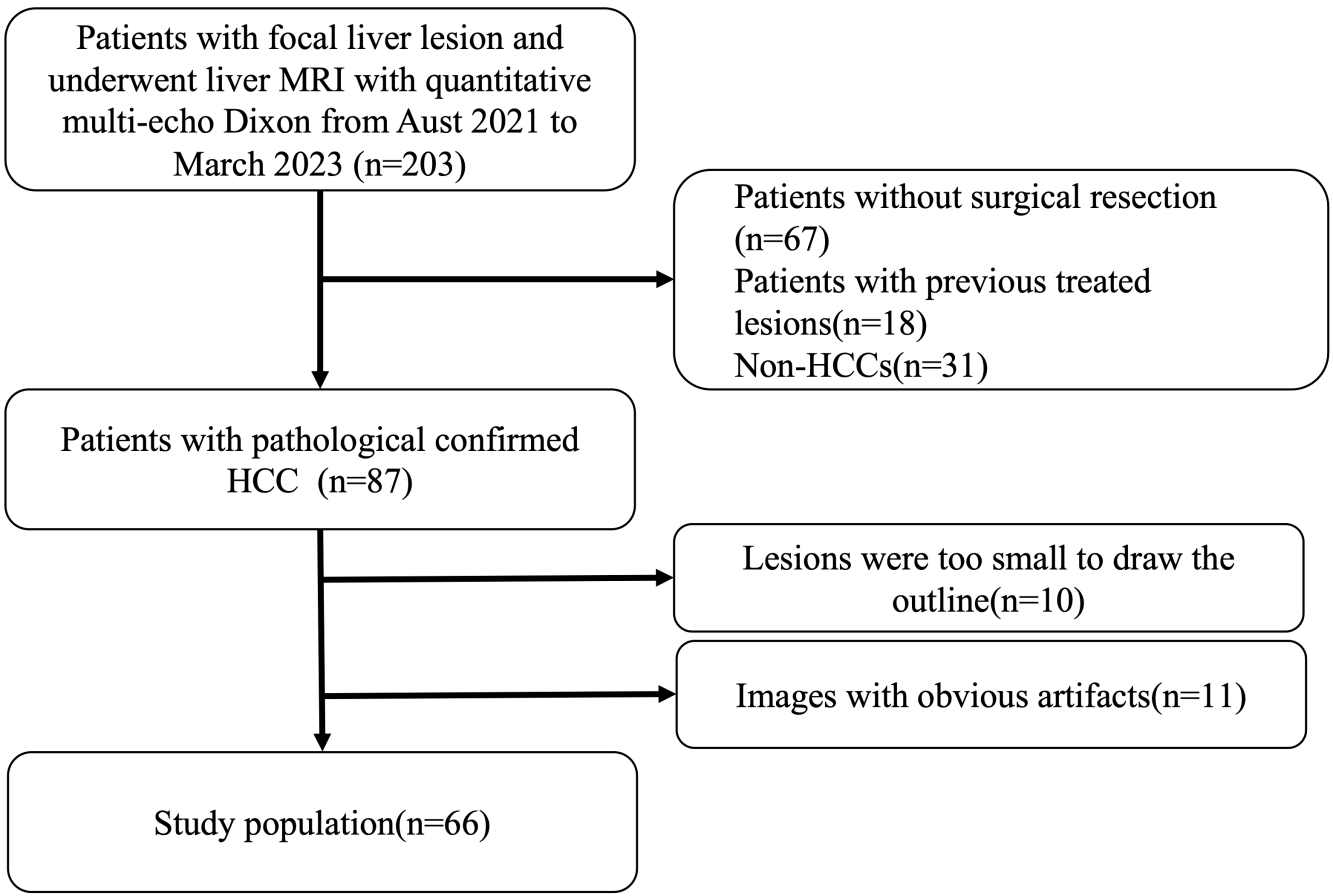
Patient selection. We enrolled 203 consecutive patients who were suspected or known of hepatocellular carcinoma clinically or at previously performed ultrasonography or computed tomography underwent dynamic enhanced liver MRI and quantitative multi-echo Dixon from August 2021 to March 2023 in this study. Patients who had undergone non-surgical treatments (n=67), had previous treated HCCs (n=18), had histopathologically proven non-HCC (n=31), encountered analysis difficulties due to small lesion size (n=10) or apparent artifacts in fat fraction mapping and R2* mapping(n=11) were excluded. And a final cohort of 66 patients with 66 lesions were enrolled.

We enrolled 203 consecutive patients who were suspected or known of hepatocellular carcinoma clinically or at previously performed ultrasonography or computed tomography underwent dynamic enhanced liver MRI and quantitative multi-echo Dixon from August 2021 to March 2023 in this study. We excluded patients who (1) had undergone non-surgical treatments (n=67); (2) has previous treated HCCs (n=18); (3) had histopathologically proven non-HCC (n=31); (4) encountered analysis difficulties due to small lesion size (n=10) or apparent artifacts in fat fraction mapping and R_2_* mapping(n=11). A final cohort of 66 patients with 66 lesions were analyzed. The study flowchart of patient selection is presented in **Figure 1**.

Clinical information including demographics, causes of liver disease and laboratory data (total bilirubin, alanine transaminase, aspartate transaminase, albumin, serum alpha-foetoprotein) were collected.

### MRI protocol

All MRI examinations were performed using the same protocol at 3.0T (MAGNETOM Skyra, Siemens, Germany) with an 18-channel phased-array coil and the spine coil with feet first and supine position. After conventional MRI sequences include the coronal T2-weighted Half-Fourier acquisition single-shot turbo spin-echo sequence (HASTE), the axial T1-weithted dual-echo sequence, the quantitative multi-echo Dixon was acquired in a single 20-second breath-hold. The detailed scanning parameters were as follows: repetition time (TR) = 9.00 msec, echo time (TE) =1.05/2.46/3.69/4.92/6.15/7.38msec, field of view (FOV) = 450 mm × 450 mm; slice thickness = 3.5mm, voxel size = 1.4 × 1.4 × 3.5mm; Flip angle= 4°; matrix size=160 ×95, bandwidth=1080Hz/Px, and an acceleration technique of CAIPIRINHA was employed with the acceleration factor was 2. A Levenberg-Marquardt nonlinear fitting was then utilized to fit the magnitude of the complex signal of the multi-echo data. Inline reconstruction was performed by addressing confounding factors that included magnetic field inhomogeneity, eddy currents, T1 bias, T2* decay, and spectral complexities.

### Imaging analysis

**Figure 2:**
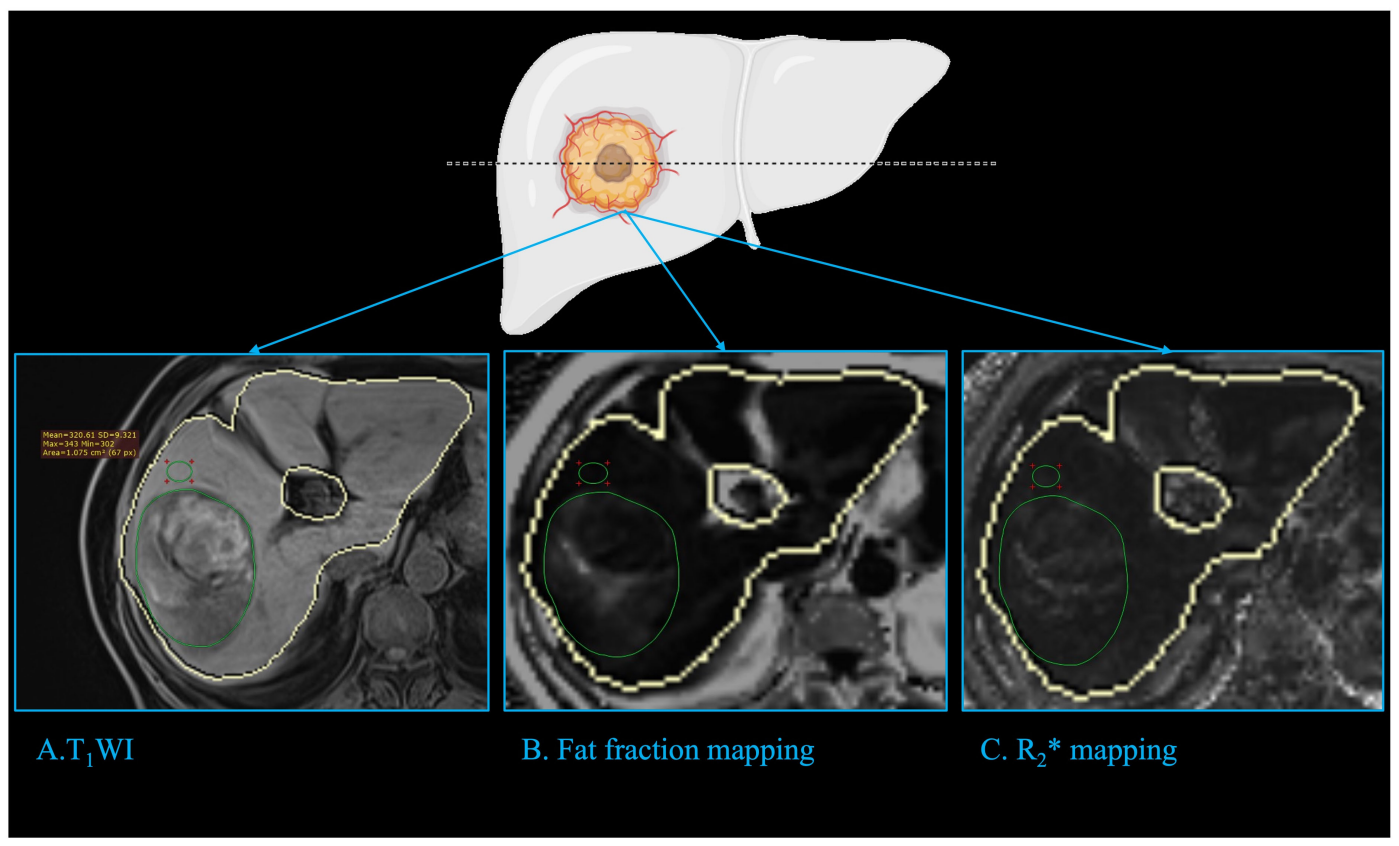
Imaging analysis. The fat fraction mapping and R_2_* mapping was automatically calculated with the liver automatically delineated (closed yellow curve). The ROI of tumor (green circle) was delineated in the maximum plane of the intatumoral fat or the tumor if no significant fat was depicted in fat fraction mapping, and was delineated in the T_1_WI **(A)** and copied to fat fraction mapping **(B)** and R_2_* mapping **(C)**. The ROI of the peritumor liver (green circle) was delineated in the peritumoral region of the tumor with a circle of about 1cm^2^, which avoided the main vessels and bile duct. ROI: region of interest.

The fat fraction mapping and R_2_* mapping was automatically calculated without the need for further post-processing. The region of interest (ROI) of the tumor, peritumoral regions, and liver were manually drawn. The ROI of the tumor was delineated in the maximum plane of the intatumoral fat or the maximum plane of tumor if no significant fat was depicted in fat fraction mapping and was delineated in the T1WI and copied to the fat fraction mapping and R_2_* mapping (**Figure 2**). The ROI of the peritumor liver was delineated in the peritumoral region of the tumor with a circle of about 1cm^2^ which avoided the main vessels and bile duct. The fat fraction of liver was the average of 3 different ROIs randomly delineated in the liver with 3 circles of about 3cm^2^, and avoided the main vessels and bile duct.

### Histopathological analysis

Data of tumor pathologic factors including size, tumor differentiation based on Edmondson-Steiner grade, MVI, satellite nodule and expression of Ki67 were obtained from pathology reports. MVI was defined as the presence of tumor cells in the vessels of surrounding liver tissue which were lined by the endothelium that was visible only on microscopy. The positive cellular index of Ki67 was evaluated by a trained pathologist and described according to the presence of nuclear staining regardless of the intensity of staining. The percentage of Ki67 was determined by counting 1000 cells/slide at 100x magnification in five randomly selected fields.

### Statistical analysis

Continuous variables were reported as means and standard deviations or medians and interquartile ranges. Categorical variables were displayed as percentages and numbers. The differences between groups were assessed using Mann-Whitney U test or Chi-squared test because data were non-normally distributed. The relationship between MRI parameters and Ki67 was assessed through nonparametric Spearman correlation coefficients. ROC analysis was performed if the MRI parameters showed statistically significant differences between the two Ki67 groups. A two-sided P-value less than 0.05 was considered statistically significant. All statistical analyses, utilizing SPSS software (version 26.0) and R statistical software (version 4.1.2), included t-tests, paired t-tests, and Mann–Whitney U tests for continuous variables, and the chi-square test for categorical variables. ROC analysis was performed using the “pROC” and “ggplot2” packages.

## Results

### Clinical characteristics

Of the 66 patients (mean age, 55.59 ± 8.99 years, 55(83.33%) males), 60 patients were infected with HBV, while 4 patients were infected with HCV and 2 patients have pathology proved chronic hepatitis change and no history of HBV, HCV, ASH, NASH and alcohol abuse. Furthermore, 18 (27.27%) HCCs were well-differentiated, while 17 (25.76%) patients had moderately differentiated HCCs and 31 (46.97%) HCCs were poorly differentiated. More details of patients are given in **Table 1**.

**Table 1:** Characteristics of the study population.

	All	Ki67%<=30	Ki67>30	P
Number	66	39	27	
Age ^a^	55.59(8.99)	56.23(8.56)	54.67(9.67)	0.492
Sex				0.737*
Female	11	7	4	
Male	55	32	23	
HBV	53	35	25	0.692*
HCV	4	2	2	0.703*
TB(umol/L) ^b^	11.95(8.57-14.95)	12.70(8.32-15.80)	13.40(9.20-14.90)	0.514**
ALT(U/L) ^b^	22.50(16.75-33.75)	22.00(18.00-30.50)	25.00(14.00-38.00)	1.000**
AST(U/L) ^b^	24.50(20.00-34.50)	23.00(18.00-33.00)	26.00(21.00-37.00)	0.855**
ALB(g/L) ^a^	39.24(3.08)	38.89(2.87)	39.74(3.34)	0.278
sAFP(ng/ml) ^b^	80.63(6.73-661.55)	9.40(3.85-260.60)	301.80(77.70-1210.00)	0.012**
Diam(mm) ^b^	43(24-83)	42.50(21.00-77.00)	46.00(25.00-96.00)	0.324**
Differentiation				0.001*
Well	18(27.27%)	16(24.24%)	2(3.03%)	
Moderately	17(25.76%)	12(18.18%)	5(7.58%)	
poorly	31(46.97%)	11(16.67%)	20(30.30%)	
MVI				<0.001*
Positive	18	3	15	
Negtive	48	36	12	
Satellite	0	0	0	

Except where indicated, data are absolute frequencies.

a Data are means (standard deviation).

b Data are medians (interquartile range).

HBV, hepatitis B virus; HCV, hepatitis C virus; TB, total bilirubin; ALT, alanine transaminase; AST, aspartate transaminase; ALB, albumin; sAFP, serum alpha-foetoprotein; Diam, diameter.

*Chi-squared test.

**Mann-Whiney U test.

### Fat fraction and R_2_* of tumor and liver

The HCCs have a median intratumor fat fraction (tFF) of 5.24% (IQR 1.88% - 8.52%) and was higher than the median fat fraction of peritumor liver (5.24% vs 3.51%, P=0.012). Of the 66 HCCs, 42 (63.64%) had an tFF of less than 5%, 13 (19.69%) had an intratumor fat fraction of more than 5% and less than 10%, and 11 (16.67%) had an intratumor fat fraction of more than 15%. The fat fraction of peritumor was not significantly different from fat fraction of liver (3.51% vs 3.54%, P =0.739). Of the 66 patients, 9 (13.64%) had fatty liver with a liver fat fraction of more than 5% (15). The fat fraction of tumor had no significant difference between the fatty liver patients and nonfatty liver patients (4.32% vs5.38%, P=0.502).

The median R_2_* of HCC was 38.55s^-1^ (IQR 26.40-47.81 s^-1^) while that of the peritumor liver was 51.40 s^-1^(IQR 42.75-69.82 s^-1^). The R_2_* of the tumor was significantly lower than that of the peritumor liver (38.55s^-1^ vs 51.40s^-1^, P<0.001).

### Relationship of fat fraction and R_2_* with the Ki67

The spearman’s correlation analysis indicated that the tFF was negatively correlated with the expression of Ki67 (r=-0.306, P=0.012). The correlation analysis showed that the fat fraction of the liver, R_2_* of the tumor and R2* of the liver were not significantly correlated with the Ki67 of the tumor (P>0.05), as shown in **Table 2**.

**Table 2:** Spearman’s correlation analysis of MRI and Ki67.

	r	P
tFF	-0.306	0.012
FF	0.037	0.770
tR_2_*	0.132	0.290
R_2_*	0.007	0.954

tFF, fat fration of tumor; FF, fat fraction of peritumoral liver; tR_2_*, R_2_* of tumor.

**Figure 3:**
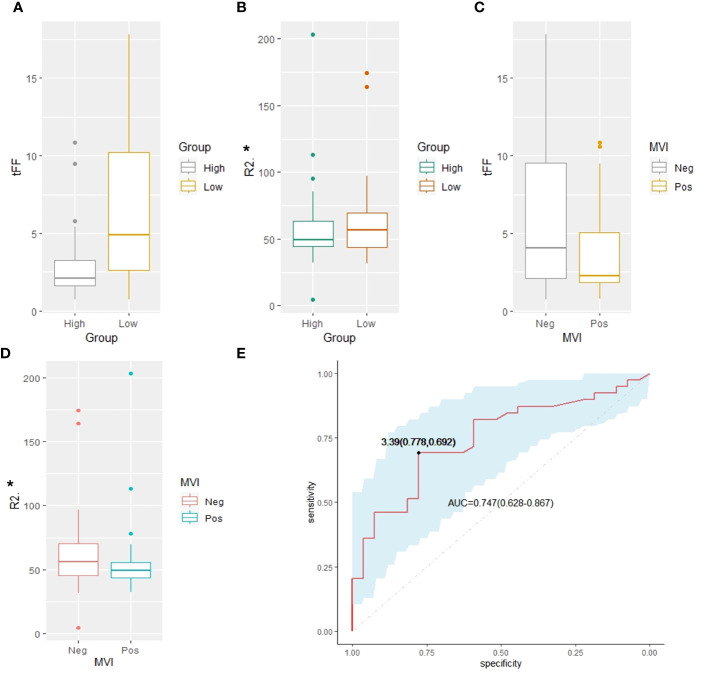
Comparison analysis. **(A)** The tFF of the high Ki67 was lower than that of the low Ki67 group (2.10% vs 4.90%, P=0.001). **(B)** The R_2_* of the high Ki67 was lower than that of the low Ki67 group (49.60 s^-1^ vs56.90 s^-1^, P=0.003). **(C)** The tFF in MVI negative group had no significant difference from that of the MVI positive group (4.05% vs 2.25%, P=0.087). **(D)** The R_2_* of the tumor in MVI negative group had no significant difference from that of the MVI positive group (36.60s^-1^ vs 39.05s^-1^, P=0.194). **(E)** The ROC analysis demonstrated that the tFF of the tumor can distinguish the Ki67<=30% group with an AUC of 0.747(95%CI 0.628-0.867) and cut-off of 3.39% (sensitivity 77.8%, specificity 69.2%). tFF, the fat fraction of the tumor; MVI, microvascular invasion; ROC, receiver operating characteristic; AUC, area under the curve.

**Figure 4:**
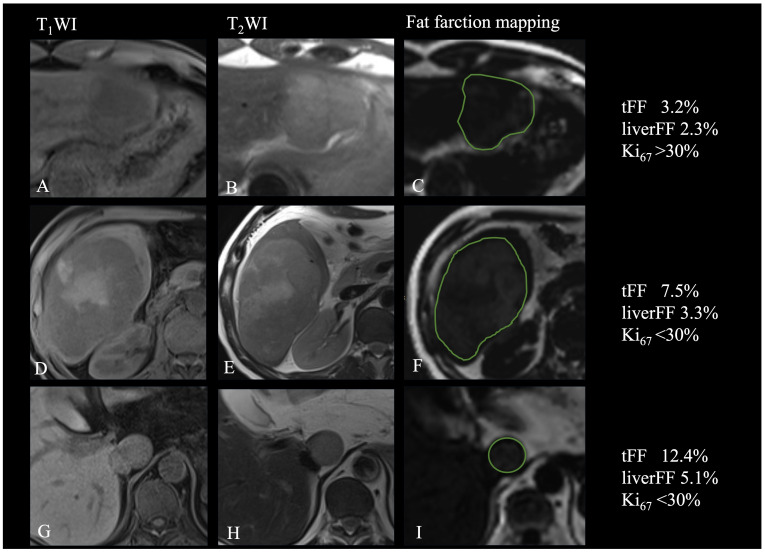
Relationship of fat fraction and R_2_* with the Ki67. **(A-C)** Patient with focal fat and high expression of Ki67. **(D-F)**. Patient with diffuse fat in mass and low expression of Ki67. **(G-I)** Patient with diffuse fat in small lesion and low expression of Ki67.

The median Ki67 of the 66 HCCs was 27.5% (IQR 10.0%-50.0%), with 39 HCCs had less than 30.0%. The tFF was higher in the Ki67<=30% group than the Ki67>30% group (4.90% vs 2.10%, P=0.001), and the R_2_* of the liver was higher in the Ki67<=30% group than the Ki67>30% group (56.90 s^-1^ vs 49.60 s^-1^, P=0.003), as shown in **Table 3** and **Figure 3**. The ROC analysis demonstrated that the tFF could distinguish the Ki67<=30% group with an AUC was 0.747(95%CI 0.628-0.867) and a cut-off of 3.39% (sensitivity 77.8%, specificity 69.2%), as shown in **Figure 4**. The R_2_* could not be used to distinguish the Ki67<=30% group (AUC 0.553, 95%CI 0.409-0.697, P=0.469).

**Table 3:** Comparison of MRI between the low Ki67 and high Ki67 groups.

	Ki67%<=30	Ki67>30	P ^a^
Number	39	27	
tFF (%)	4.90(2.50-10.50)	2.10(1.60-3.30)	0.001
FF (%)	3.00(1.35-4.80)	3.00(2.10-3.60)	0.634
tR_2_* (s^-1^)	39.20(26.60-49.10)	38.30(25.80-47.60)	0.469
R_2_* (s^-1^)	56.90(41.20-69.80)	49.60(42.90-69.90)	0.003

Data are medians (interquartile range).

a Mann-Whiney U test.

tFF, fat fration of tumor; FF, fat fraction of peritumoral liver; tR_2_*, R_2_* of tumor.

### Relationship of fat fraction and R_2_* with the MVI

Of the 66 patients, 48 (72.73%) had no MVI in the peritumoral liver, while 18 (27.27%) had MVI in at least one vessel in the peritumoral region. The fat fraction and R_2_* of the tumor in MVI negative group were not significantly different from those in the MVI positive group (4.05% vs 2.25%, P=0.087; 36.60s^-1^ vs 39.05s^-1^, P=0.194). The fat fraction and R_2_* of the liver in the MVI negative group were also not significantly different from those in the MVI positive group (3.00% vs 2.90%, P=0.784; 55.80s^-1^ vs 49.15s^-1^, P=0.227), as shown in **Table 4**. The frequency of MVI was not significantly different between the high tFF HCCs and the low tFF HCCs (25.00% vs 30.00%, P=0.650).

**Table 4:** Comparison of MRI between the negative and positive MVI groups.

	MVI (-)	MVI (+)	P ^a^
Number	48	18	
tFF (%)	4.05(2.02-9.58)	2.25(1.75-5.70)	0.087
FF (%)	3.00(1.46-4.74)	2.90(2.05-3.66)	0.784
tR_2_* (s^-1^)	36.60(24.03-48.72)	39.05(31.94-47.58)	0.194
R_2_* (s^-1^)	55.80(43.62-71.10)	49.15(41.02-59.82)	0.227

Data are medians (interquartile range).

a Mann-Whiney U test.

MVI, microvascular invasion; tFF, fat fration of tumor; FF, fat fraction of peritumoral liver; tR_2_*, R_2_* of tumor.

## Discussion

This study estimated the relationship between the proliferation marker Ki67 of HCC and the fat fraction of the tumor, as determined by the MRI quantitative multi-echo Dixon. Furthermore, the ROC analysis highlighted the fat fraction of the tumor as a good discriminator of HCCs with Ki67<=30%. Thus, we have successfully validated the fat fraction as an accurate, noninvasive, and *in vivo* biomarker of proliferation for HCCs.

The tumor proliferation of HCC can be non-invasively evaluated with MRI. Chen at al. reported a strong relationship of Ki67 with Gd-EOB-DTPA-enhanced MRI in HCC (16). And Jing at al. indicated that the expression of Ki67 was associated with apparent diffusion coefficients (17). Gd-EOB-DTPA-enhanced MRI and apparent diffusion coefficient is an advanced MRI technique, which needs special contrast media and advanced post-operative software. And these two techniques were used to evaluate the tumor proliferation from the perspective of uptake of specific contrast agents and tissue microstructure. Furthermore, Hu et al. estimated the value of viscoelasticity measured by MRI elastography for prediction of Ki67 expression (18). Our study revealed similar result that the MRI quantitative multi-echo Dixon was a good discriminator of Ki67, which is a completely different MRI technique that mainly reflects fatty changes within the tumor. To our knowledge, it was the first to evaluate the proliferation of HCC using the fat quantification of MRI.

The intra tumoral fat is an important feature of HCC (19) and represents focal hypoxia due to the transition of blood supply from portal vein to hepatic artery with insufficient neovascularization. Intra tumoral fat was found in both early stage and progressed HCCs. Our study demonstrated that 13 (19.69%) patients had an intratumor fat fraction of more than 5% and less than 10%, and 11 (16.67%) had an intratumor fat fraction of more than 15%, with most patients having chronic HBV and HCV infection, which suggested that intra tumoral fat was not only found in patients with fatty liver. Furthermore, our study reveals a negative correlation between fat fraction and Ki67, suggesting that intra-tumoral fat serves as a marker of tumor proliferation. The most possible hypothesis may be that the intra tumoral fat was caused by dysregulated lipogenesis that was due to clonal proliferation in proliferative HCCs (20). And the intracellular lipid droplets broke down if energy was deficient in proliferative tumors (21). Hence, the intra tumoral fat can also be found in proliferative HCCs and the fat fraction may be less than that in less proliferative HCCs. Previous studies have also suggested that poorly differentiated HCC is prone to have focal steatosis (5), which may explain the relative low-fat fraction observed in high Ki67 group in our quantitative measurement study. This study provides a quantitative method for measuring fat fraction and demonstrates its potential as an imaging marker of tumor proliferation.

Some studies have reported that increased fat content may correlated with improved prognosis (14, 22). The mild accumulation of fat may reflect changes in the microenvironment of hepatocellular carcinoma tumors that reduce tumor aggressiveness. Tani et al. also demonstrated that the peritumoral fat content, as identified by MRI, correlates with prognosis of breast cancer (23). This study demonstrates the potential of fat fraction as an imaging marker of tumor proliferation, which is a prognostic factor in HCC. Consequently, the fat content in hepatocellular carcinoma may affect the response to treatment, such as resection, chemotherapy, or radiation therapy (22). In summary, fat content is associated with outcomes in patients with HCC and has important clinical implications. Through further research and validation, we can better use this indicator of fat fraction based on MRI to guide the treatment and management of HCC patients, and it may also have application prospects in other types of cancer.

Additionally, our study finds that the fat fraction of the tumor does not significantly differ between patients with fatty liver and non-fatty liver, despite a previous study indicating a higher median fat fraction in HCCs with liver steatosis (3). It’s worth noting that steatotic and steatohepatitic HCCs, which often exhibit diffuse fat in the mass (24), are closely associated with underlying fatty liver disease and metabolic syndrome (25). Most of the patients with fatty liver or without fatty liver in this study were accompanied by chronic HBV or HCV infection. Chronic hepatitis is suggested to have an influence on metabolic changes (26), potentially leading to fatty liver development. Therefore, the observed fatty liver in our study may be attributed to chronic hepatitis rather than non-alcoholic fatty liver disease or metabolic syndrome, explaining the lack of significant differences in tumor fat fraction between the two groups.

Numerous studies have been devoted to the preoperative prediction of MVI and the results are still uncertain. In our present study, the fat fraction determined by MRI quantitative multi-echo Dixon did not exhibit significant difference between the MVI-negative and MVI-positive groups. Some researchers have indicated that the intra tumoral fat may suggest a lower risk of MVI (7), which was partially explained by the relationship of intra tumoral fat and lower histological grades. However, not all studies support this finding (27). Further investigations, such as subclassification analysis of MVI number and location (28), or advanced analyses of intra-tumoral fat using texture analysis or radiomics (29), may enhance our understanding of this relationship.

This study has some limitations. First, it was a retrospective, single-center research with a small number of HCC patients accompanied with HBV, HCV infection or pathology proved hepatitis. However, the present study was the first to evaluate the proliferation of HCC using the fat quantification of MRI. Therefore, it will be necessary to confirm the results through prospective, multicenter study with a larger number of patients. Second, the Ki-67 index has traditionally served as a biomarker indicating tumor proliferation, while recent studies has classified HCC proliferation through diverse molecular analyses and genomic profiling. Third, we did not assess the influence of ROI sampling strategies on measurement and reproducibility of quantitative multi-echo Dixon. Previous studies have indicated that the reproducibility and repeatability of measurements were improved as much area of the ROI (30). Therefore, the ROI of the tumor was delineated in the maximum plane of the intatumoral fat or tumor in this study. And the fat fraction based on MRI has been extensively explored in the literature and is considered a stable and non-invasive tool compared to pathology. Forth, the fat fraction in the maximum plane of the intatumoral fat or tumor was used to analyze, and the distribution of intra tumoral fat was not evaluated. As the distribution of fat is another important feature for the diagnosis of steatotic or steatohepatitic HCCs, we hypothesize that the quantification and distribution of fat could offer greater clinical insight for prognosis and treatment. Thus, we intent to investigate further how fat quantification and radiomic or machine learning features will influence patient prognosis and treatment outcomes in subsequent studies. Lastly, this study did not investigate how fat quantification affects patient outcomes due to the limited follow-up time. Previous studies have shown that fat sparing in solid mass was potential marker to evaluate the survival with favorable performance and discriminator of complete response under TACE treatment (14). Therefore, a further validation study is needed to confirm the prognostic value of fat fraction.

## Conclusion

In conclusion, fat fraction quantification based on quantitative multi-echo Dixon can effectively differentiate low-proliferation HCCs, although it may not be suitable for diagnosing MVI. Fat fraction quantification holds promise as a potential prognostic predictor of treatment option.

## Data availability statement

The original contributions presented in the study are included in the article/supplementary material. Further inquiries can be directed to the corresponding author.

## Ethics statement

The studies involving humans were approved by Ethics Committee of Tongji Hospital, Tongji Medical College, Huazhong University of Science and Technology. The studies were conducted in accordance with the local legislation and institutional requirements. The ethics committee/institutional review board waived the requirement of written informed consent for participation from the participants or the participants’ legal guardians/next of kin because This was a retrospective study and the patient information was encrypted during the study.

## Author contributions

MH: Conceptualization, Data curation, Formal analysis, Funding acquisition, Investigation, Methodology, Project administration, Resources, Software, Supervision, Validation, Visualization, Writing – original draft, Writing – review & editing. FZ: Investigation, Data curation, Formal analysis, Methodology, Project administration, Software, Validation, Writing – original draft. ZL: Data curation, Formal analysis, Investigation, Methodology, Project administration, Software, Validation, Conceptualization, Resources, Supervision, Writing – review & editing. YL: Conceptualization, Data curation, Formal analysis, Investigation, Methodology, Project administration, Writing – review & editing, Funding acquisition. JL: Conceptualization, Data curation, Formal analysis, Funding acquisition, Investigation, Methodology, Project administration, Writing – review & editing, Resources. ZW: Conceptualization, Data curation, Formal analysis, Investigation, Methodology, Project administration, Writing – original draft. LM: Investigation, Methodology, Project administration, Resources, Validation, Writing – review & editing. GC: Investigation, Methodology, Project administration, Writing – review & editing, Conceptualization, Data curation, Formal analysis, Software. XH: Conceptualization, Investigation, Writing – review & editing, Resources, Supervision.
