# Corrigendum: Fat fraction quantification with MRI estimates tumor proliferation of hepatocellular carcinoma

**DOI:** 10.3389/fonc.2024.1418905

**Published:** 2024-05-22

**Authors:** Mengqi Huang, Fan Zhang, Zhen Li, Yan Luo, Jiali Li, Zixiong Wang, Liya Ma, Gen Chen, Xuemei Hu

**Affiliations:** Tongji Hospital, Tongji Medical College, Huazhong University of Science and Technology, Wuhan, Hubei, China

**Keywords:** hepatocellular carcinoma, fat fraction, proliferation, MVI, MRI

In the published article, there was an error in the correspondence, as Gen Chen and Xuemei Hu are our co-corresponding authors.

The authors apologize for this error and state that this does not change the scientific conclusions of the article in any way. The original article has been updated.

